# Relation between Facial Neuralgia and Dental Irritation

**Published:** 1886-03

**Authors:** J. M. Ackland

**Affiliations:** Southernhay, Exeter


### RELATION BETWEEN FACIAL NEURALGIA AND DENTAL IRRITATION.

BY

J. M. Ackland, M.R.C.S., L.D.S. Eng.,

*Southernhay, Exeter.*

---

In the December number of this Journal is a paper by Mr. H. Boyle Runnalls, on the subject of Facial Neuralgia, wherein he gives his experience, as a surgeon in general practice, of cases of so-called facial neuralgia, where a great amount of medicine has failed to give relief, but in which attention to the teeth has been rewarded by a speedy cure. I can endorse much of what he says from cases which have come under my care as a dental surgeon, both in private and hospital practice.

One has only to remember the important part played by the fifth nerve, with its widespread divisions and branches, in the nervous supply of the face and deeper tissues, also that many of the ultimate ramifications of this nerve end in the very teeth themselves, to see how easily these dental organs can, when diseased, produce pain in the surrounding parts. Again, how very intimately this nerve is connected with others by its ganglia, each of which has its sensory, molar, and sympathetic root.

Facial neuralgia, arising from the teeth, may be either *reflex*, *direct*, or *complex*.

In the *first* case, some portion of the nervous system receives an exaltation of function from the tooth nerve.

In the *second*, some contiguous nerves are involved by the spread of inflammation from diseased teeth.

In the *third*, both would be entailed in a mixed and uncertain proportion.

In facial neuralgia, it is generally found that pressure on the points of exit of branches of the affected nerve from their bony canals, such as the infra-orbital, mental, &c., is extremely painful.

One of the most constant sources of neuralgia is the wisdom-tooth, which so often becomes impacted, or from other causes unerupted; and thus, from pressure on the nerves, &c., produces the most intense pain. This is, in many instances, relieved by free incisions over the buried tooth, whilst in other cases further treatment is necessary. I remember being consulted, some six months ago, by a young married lady, twenty-six years of age, whose lower wisdom-teeth were impacted; and as no other treatment gave any relief, and the second molars were in a good condition, I, after no little difficulty, extracted both wisdom-teeth under chloroform. The neuralgic pains which had hitherto troubled her, stopped at once.

The wisdom-teeth of the lower jaw very frequently give rise to otalgia, and I could cite not a few instances in which their extraction has effected a complete cure. Many cases of so-called earache are, doubtless, examples of neuralgia affecting the auriculo-temporal division of inferior maxillary nerve.

Odontalgia, or toothache, and the neuralgic pains which often accompany it, finds its most frequent cause in exposure of, or pressure on, the tooth pulp; and what is often embarrassing, even to the most experienced dental surgeon, is the transference of the seat of pain so frequently met with, often to the fellow tooth of the opposite jaw, and now and then to more remote parts of the body, which Bunton accounts for by the irritation, conveyed to the centres of sensory branches of the fifth pair, being transmitted to those of vaso-motor centres, from which they are not far distant, and thus the blood-vessels in distant parts become affected.

The formation of secondary dentine (and hence pressure on the pulp), and exostosis of the fang, give origin to the most acute neuralgic pain. In these cases the pain is reflected from the spot of irritation over a large nervous area; but the tooth containing the offending growth is usually perceptible by tenderness or elongation. Where it arises from an exostosis, it is apt to be repeated with several teeth. These cases are not at all uncommon, and are often made the subject of unavailing medical treatment for a long time.

Again, overcrowding of the teeth frequently causes facial neuralgia; and among the nerves of special sense which have been affected by reflex nervous action from tooth-irritation are the auditory and optic.

Indeed, time and space would fail me in mentioning the various neuralgic affections which have been proved conclusively to arise from some affection of the teeth; and whilst I am convinced that many patients are allowed to suffer longer than they ought, because their teeth are not examined and attended to, I am equally certain that many serviceable teeth have been sacrificed without alleviating in the slightest degree the ofttimes terrible agony of facial neuralgia. It must not be forgotten that any irritation to a portion of a sensory nerve in its passage to the brain will cause pain, which is referred to the peripheral extremities of the fibres irritated; hence there may apparently be pain in teeth which are in no way the cause of it. Sometimes, however, the extraction of a tooth will temporarily stop the pain, which soon returns as bad, if not worse, than before. It is probable that in these cases the extraction of the tooth has led to the stretching of the nerve, and thus afforded relief for a while.

Mr. Runnalls gives as his experience, that *every* case of "neuralgia," not due to a nerve implicated by a growth, carious bone, or scar, is the result of dental irritation; but I think he has omitted to name one all-important factor.

The late Dr. Anstie, in his excellent work on *Neuralgia and its Counterfeits*, says "it is universally the case that the condition of the patient at the time of the first attack is one of *debility*;" and here undoubtedly is a cause of facial neuralgia, in which the teeth, be they ever so affected, may play a very secondary part, as also in those who are the subjects of rheumatism, gout, &c.

Some months ago I was earnestly entreated by a lady to extract an upper molar tooth, which had a small stopping in it; but after trying the usual tests of heat and cold, percussion, &c., I felt convinced that the tooth ought not to be removed then at any rate, more especially as the patient, who had been anxiously watching a sick daughter for many nights, was much below par. Grain doses of the sulphate of quinine and a few glasses of port wine entirely removed the pain, which the patient said arose from the tooth.

Dr. Charlton Bastian, on Diseases of the Nervous System, in Quain's *Dictionary of Medicine*, shows, in discussing the etiology and pathology of the subject, how an altered condition of the blood, say in anæmia, may produce a diminution, exaltation, or other perverted action in a nerve, especially in those of a neurotic habit.

Whilst Dr. Buzzard, on the subject of Tic-Doloureux, in the same work, says: "Cold wind, especially with a moist atmosphere, has an undoubted influence in starting neuralgia of the fifth pair, the unprotected condition of the face explaining, probably, its peculiar liability to be so attacked. There appears reason to think, however, that when damp, with cold, excites an attack of neuralgia, there must be at the same time a peculiar condition of the system. . . . Such a condition is probably of a rheumatic or gouty nature, and the cold seems to start a subacute neuritis in the sheath of the nerve." And cases are common in which facial neuralgia occurs more or less regularly in females at the menstrual periods or during pregnancy.

Mr. Runnalls mentions one or two cases of facial neuralgia, then goes on to talk of amaurosis, and finally gives instances of pain arising from teeth which had been stopped. Possibly (and this sometimes happens to the best of practitioners, in spite of all their precautions) a filling had been placed too near the nerve, resulting in the death of the latter, or a small particle of septic matter had escaped attention in the preparation of the tooth. Be that as it may, the pain is, in the majority of cases of this description, instantly relieved by a very simple operation, that of drilling a small hole at the neck of the tooth, underneath the edge of the gum, into the pulp chamber, and so giving free exit to the gases and fluids which have been generated by the death of the pulp. In other cases the filling may be taken out, the nerve canal thoroughly cleansed, treated, and refilled.

Cold, violence, rheumatism, or syphilis may cause a similar result in a perfectly sound tooth, an embolism being formed in the nutrient vessels of the pulp.

This state of things is generally associated with peri-odontitis, or inflammation of the alveolo-dental membrane (for branches of the same vessels supply pulp inside and membrane out), the pathology, &c., of which would be too long to enter into here, even were it the place for so doing. Suffice is it to say that unless this condition is diagnosed and successfully treated it passes through various stages, and ultimately ends in abscess, as it appears to have done in a case mentioned by Mr. Runnalls.

Now this may often be relieved by giving the pus vent, either through the tooth, as mentioned above, or where the abscess points (generally on outside of alveolus), and then treating in various ways.

These troubles often occur in connection with front teeth, and there is frequently no more reason for sacrificing the tooth, in spite of its bony surroundings, than there is, in the majority of cases of whitlow, for amputating the finger. The relief experienced when an exit is made for the pent-up fluids is as complete in one case as the other. Some few weeks since a medical friend called late one night, requesting me to do anything I could to relieve him of the terrible pain he had been in for many days, suffering in nearly all his front lower teeth, as the result of exposure during the late severe weather. All the teeth were sound, but his sufferings were so great that he offered to lose any of them to be free from pain. Although he had allowed some considerable time to elapse before coming to me, I prevailed upon him to try a little longer. Nothing, however, seemed able to arrest the inflammation, which ultimately ended in the death of the nerve of the lower right central tooth, the tension, and with it the pain, in which was immediately relieved by a hole drilled as before mentioned.

I trust I shall not be considered egotistical. This is but one of many instances, but their treatment seems to me to be purely a matter of dental surgery; and my only object in mentioning it is, to show that cases of the kind are to be treated easily and successfully, and yet such a wholesale extraction avoided. Difficult as it is, in many instances, to diagnose the cause of the pain, a well-marked case of facial neuralgia, unaccompanied by any other trouble, differs in many ways from that of a collection of pus as described by Mr. Runnalls, or, in other words, an abscess. The history, as well as the appearance, of the two cases would vary considerably, and this would help one to diagnose the cause of the mischief. On the other hand, there are, of course, cases just as difficult as these would be easy, and in which extraction is an absolute necessity. No one would deny the *right* of any surgeon to take upon himself to decide for his patients what teeth are to be preserved and what extracted, and without further ado removing the latter; but many, I am sure, would question the wisdom of such a proceeding, especially in these days of conservative surgery, no one branch of which has made more advancement than that of dental surgery.

Mr. Runnalls remarks, and very truly, that the teeth of the present generation are in a far worse condition than those of the last, and still seem to be degenerating. Is not this another reason why the teeth, bad as they may be, should be preserved as long as possible? Besides, what does this continual extraction mean? Merely a substitution of dyspepsia for neuralgia. In conclusion, whilst in the main I can endorse much of what this gentleman says on facial neuralgia and its connection with the teeth, his attempt to pose as a critic in dental matters must be allowed to pass for what it is worth; for no one but those who have thoroughly studied the teeth and their diseases can, in the face of the difficulties which are so constantly met with, be possibly able to give any reliable advice thereon; and in the present day there is every opportunity for patients of all classes, either in private or hospital practice, to be relieved from pain arising from dental troubles, and at the same time to have trustworthy advice as to what teeth should be sacrificed and what treated and preserved.

